# An Objective Approach to Identify Priority Rare Diseases for the Development of Solutions Reducing the Diagnostic Delay Based on French Data

**DOI:** 10.3389/fphar.2021.734601

**Published:** 2021-10-22

**Authors:** Pierre Etienne Chazal, Ségolène Aymé

**Affiliations:** ^1^ Orange Business Services SA, Orange HealthCare, Paris, France; ^2^ Paris Brain Institute, ICM, Inserm U 1127, CNRS UMR 7225, Sorbonne University, Paris, France

**Keywords:** rare diseases, diagnostic delay, clinical guidelines, orphan drugs, public health, eHealth

## Abstract

A timely diagnosis is a critical step to ensure a proper access to expert clinical management for patients. However, diagnosing rare diseases (RD) is a major challenge, as they are not only numerous but also extremely diverse in their expression and cause. This generates a long lag time between first symptoms and diagnosis, unanimously thought to be unacceptably long in many cases, and amenable to improvement. Digital technologies offer new opportunities for improving diagnosis and care in a sector with urgent needs. However, developing and testing digital solutions would only be possible for a limited number of rare diseases (RD). The approach presented here aims at proposing an objective way of defining a subset of “priority” RD to focus on for the development and test of new solutions to reduce the time to diagnosis. An approach which is relevant not only when developing and testing new digital solutions but also organizational solutions in the field of RDs. The priority RDs presented herein have been highlighted using two objective criteria: the existence of a well-defined and established standard of care management, defined as the availability of a medicinal product specifically targeting the disease; and / or the existence of authoritative clinical guidelines. Our approach, based on French data, led to the establishment of a list of 251 RD for which a delayed diagnosis would be especially detrimental for the patient. This work demonstrates the feasibility of identifying objectively a subset of RD at urgent needs for the development of solutions to reduce the delay to diagnosis, if choices have to be made, based on publicly and well-established available data. The proposed list needs to be updated and adapted to the local situation, and validated by experts to establish if the delay to diagnosis can be reduced.

## Introduction

Diagnosing rare diseases is a major challenge. Rare diseases (RDs), whose definition is based on a prevalence notion, are not only numerous (more than 7,000 are described, mostly with a genetic origin) but also extremely diverse in their expression, cause, semiology and nosology. Many RDs share symptoms with “common” diseases, making suspicion of a RD all the more complicated for non-expert practitioners. Moreover, the diagnosis remains complicated even for the best experts, despite increasing knowledge and new imaging or biological and molecular technologies.

This generates a long lag time between first symptoms and diagnosis. A delay that has been identified as a key problem to be fixed by patient organizations (Eurordis, 2009), as a timely diagnosis is a critical step to ensure proper access to expert clinical management. However, this delay is unanimously thought to be unacceptably long, and amenable to improvement if appropriate measures are undertaken.

The reasons for such a delay are diverse and cumulative. A delayed diagnosis can occur because the symptoms are nonspecific or uncommon for the specific disease, because scientific knowledge is still limited, because of a lack of required laboratory tests, or because all investigations were performed without any conclusive result. Sequencing and bioinformatics alone are insufficient to diagnose all inherited rare diseases, for example. These delays cannot be avoided at a given time point.

In contrast, the determinants of the healthcare systems contributing to delays could be addressed. Those may include health professionals’ lack of awareness and experience with RD, difficulties in referring patients to expert centers, lack of specialized centers or too distant ones, understaffed expert centers, or limited access to genomic services. Up to now, many initiatives have addressed these issues. In Europe, Orphanet was specifically established in 1997 to disseminate the information on RDs and expert resources. In 2004, the French Government adopted the first Public Health Plan for rare diseases, including the establishment of a network of expert centers in academic hospitals and many other initiatives likely to contribute to a better diagnosis of RDs (PNMR 1, 2004). A recommendation of the Council of European Ministries was adopted in 2009, urging all European countries to set up a national plan or strategy for RDs before 2014. A recommendation followed by most countries (Official Journal of the European Union, 2009; Khosla and Valdez, 2018). With the progressive availability and affordability of Next-Generation Sequencing (NGS) technologies, the debate around solution for the diagnosis of rare diseases focused on the access to sequencing technologies and on accelerating the identification of disease-causing genes by involving all undiagnosed patients in research protocols (Gahl et al., 2016; Boycott et al., 2019).

Improving the diagnosis of RDs still remains an enormous challenge for public and private actors, as it is a polymorph phenomenon, encompassing all aspects of medicine. However, today, the development of digital technologies offers genuine opportunities for progress: for patients and their caregivers, with new tools and options for dealing with their condition; for healthcare professionals with tools supporting their daily administrative, medical and research duties; for Healthcare systems, with tools to optimize care coordination. The sector of rare diseases is at urgent needs and the community is organized and dedicated enough to quickly adopt innovations that could improve patients’ quality of life.

In this context, a group of stakeholders was invited, by Sanofi France in partnership with Orange Healthcare, to identify tangible eHealth, but also organizational, solutions to reduce diagnostic delay at different stages of the diagnostic pathway. After 30 individual interviews and three workshops, the group identified 13 obstacles, sources of diagnostic delay, and suggested 14 digital-based solutions to reduce them. The outcome of this brain storming exercise was published as a white book, in 2018 (Sanofi, 2018).

During the process of deciding about the potential solutions, the issue of ways to test these solutions, was raised. It became clear that it would only be possible for a limited number of RDs, but that the prioritization could lead to major ethical tensions.

This study was conceived to explore an objective approach to prioritize RDs, considering that a delayed diagnosis is especially detrimental when an expert management, medicinal product and/or clinical guidelines, has been already proved effective. For sure, this choice does not imply that an absence of diagnosis, or a very late diagnosis, is not detrimental in the context of other diseases. Of course, it is the case for all of them. The current approach just aims at proposing a rational way of choosing RD for developing and testing digital-based pilots or organizational solutions, assuming that most of them will have to be customized for each specific disease or group of disease and/or adapted to each medical area.

Rare cancers and Rare infectious diseases were deliberately excluded as they are not considered by national RD plans or strategies adopted by most European countries due to their specificities.

## Materials and Methods

### Definitions and Sources

In an attempt to rationally define a subset of “priority” rare diseases to focus on, it was decided to concentrate on objective missed opportunities for patients, namely the availability of a medicinal product specifically targeting the disease; and/or the existence of authoritative clinical guidelines.

A targeted medicinal product was defined as a medical product with a Marketing Authorization (MA) with designation for one or more RDs (Orphan drugs and non-Orphan drugs); and products in development available as part of an Authorization for Temporary Use in France (ATU). These authorizations are given, prior to the MA granting, for the exceptional use of experimental pharmaceutical products that do not have yet MA for a targeted disease, and for patients that cannot be included in a clinical trial (ANSM, 2017a). Two open access sources of information were used: the list published by Orphanet, of Orphan (OD) and non-Orphan (NON-OD) drugs intended for RD and with a Marketing Authorization in the European Union (EU) as of July 2017 (Source #1) (Orphanet, 2017); and the list of drugs with an Authorization for Temporary Use (ATU) in France with on OD designation as of November 2017 (Source #2) (ANSM, 2017a; DGOS, 2017; EMA, 2017).

Regarding authoritative clinical guidelines, we considered the protocols elaborated either by the French National Authority for Health (Haute Autorité de Santé, HAS) or by the French Rare Disease networks (FSMR) following the methodology elaborated by the HAS. These protocols are syntheses of published good practices about a rare disease, or a group of rare diseases, followed by recommendations for follow-up and care. Their objective is to guide healthcare professionals (HCP) for an optimal diagnostic and therapeutic management. Two open access sources of information were used: the list of National Diagnosis and Care Protocols (NDCP) published by the HAS (Source #3) (World Health Organization, 2018); and the list of NDCPs written or under writing by the 23 FSMR according to their websites (Source #4) (DGOS, 2018).

Finally, the identified pathologies were matched with Orphanet nomenclature database (Source #5) (Orphanet, 2018). The detail of the information sources used in this work is available in the Supplementary Material.

### Methodology

**FIGURE 1:**
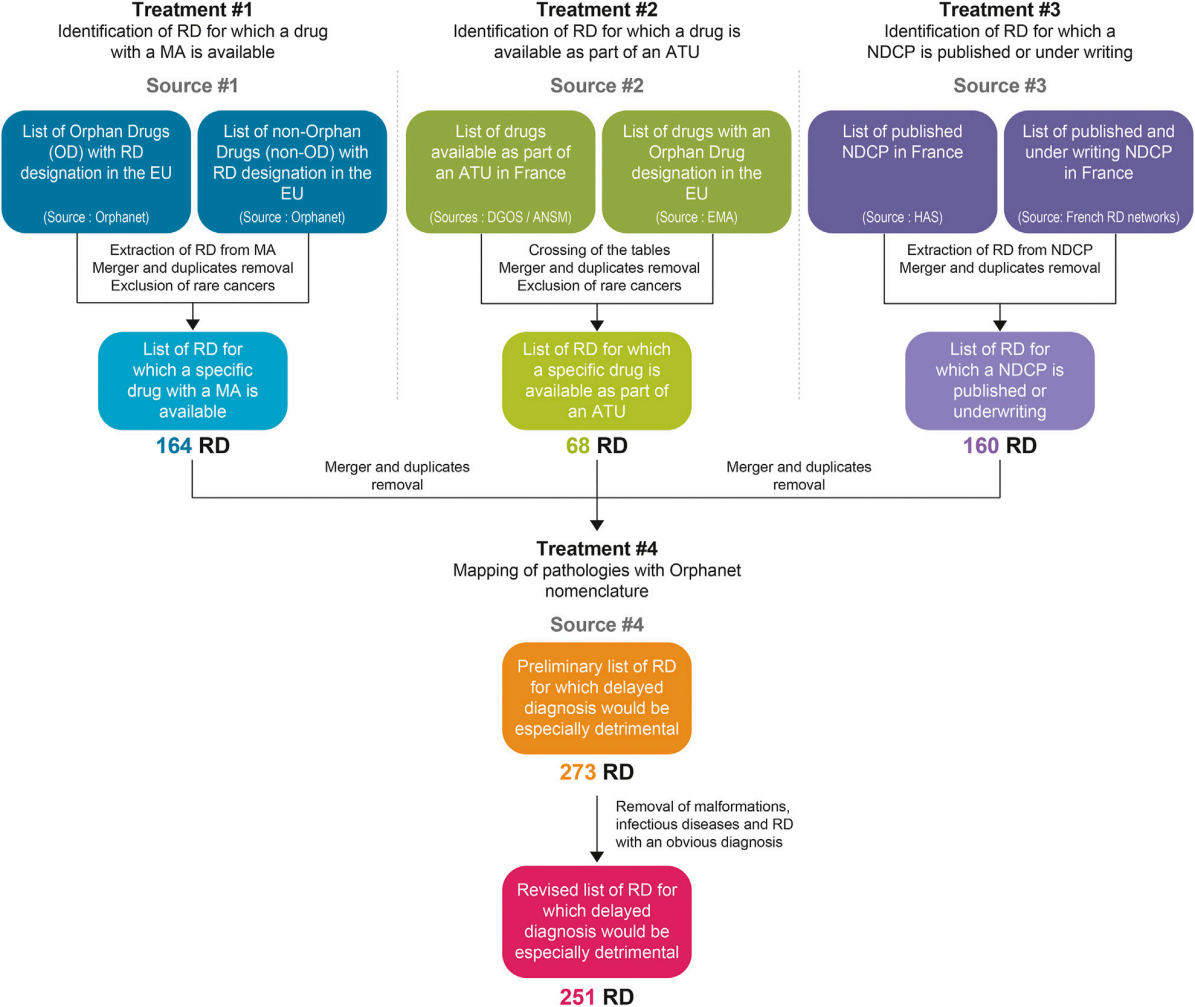
Decision-tree to identify rare diseases for which a delayed diagnosis would be especially detrimental, using existing open access sources on information, in France, on drugs intended for rare diseases and on clinical management guidelines.

A four steps methodology was designed (Figure 1):

• #1: Identification of RDs for which a commercial drug with a MA is available,
• #2: Identification of RDs for which a drug is available as part of an ATU,
• #3: Identification of RDs with a published or under writing NDCP,
• #4: Merger, duplicates removal and mapping of pathologies with the Orphanet nomenclature.

All of the treatments described below were performed using the Microsoft Excel Suite.

#### #1: Identification of RD for Which a Commercial Drug With a MA is Available

The “source #1” tables encompassed 256 drug entries: drugs with Orphan Drug (OD) designation (98 entries) and drugs without Orphan Drug (NON-OD) designation (158 entries) (Orphanet, 2017). For each drug entry, the Marketing Authorization description was manually processed to extract the names of the RD targeted, resulting in 371 “drug x RD” entries. Duplicates were removed using both Excel automatic tool then manual processing (107 duplicates merged, 264 unique RD entries remaining). Rare cancers were discarded from the final table (167 RD entries remaining–97 cancer entries discarded) as they are not considered for the production of clinical guidelines and are supported outside the rare disease networks. Conditions linked to the administration of medicinal products were also excluded: anthracycline extravasation, methotrexate toxicity and hepatitis B reinfection following liver transplantation (164 RD entries remaining–3 RD entries discarded).

#### #2: Identification of RD for Which a Drug is Available as Part of an ATU

To ensure an exhaustive listing of drugs with an ATU available in France as of November 2017, two sources (Ansm, 2017b; DGOS, 2017) were merged (281 drug entries remaining). Drug products for which an end-date of ATU was already ruled were discarded (224 drug entries remaining–57 drug entries discarded). The table was compared with the EMA Orphan drug designation table (EMA, 2017), which included all products with an ongoing application for the “Orphan Drug” status by the EMA. Given the difference of language between the sources, the two tables were compared based on the “Active Substance” (66 drug entries matched: 41 automatic matches +24 manual additional matches). The “Orphan Drug” designation which had a “withdrawn” or “negative” status were excluded (55 drug entries remaining–11 drug entries discarded). A search of the RD targeted by the 55 products was then carried out in the EMA Orphan drug designation table (column “Disease/condition”) (EMA, 2017). Duplicates were manually removed. Finally, rare cancer entries were excluded (68 RD entries remaining–6 cancer entries discarded).

#### #3: Identification of RD With a Published or Under Writing NDCP

To ensure an exhaustive listing of drugs with a NDCP, both sources (HAS, 2017; DGOS, 2018) were merged (104 NDCP entries remaining) and completed with the list of NDCP in the process of drafting and/or planned according to the FSMR websites (160 NDCP entries remaining). For each NDCP entry, the description was processed to extract the names of the targeted RD (160 RD entries).

#### #4: Merger, Duplicates Removal and Mapping of Pathologies With Orphanet Nomenclature

The three RD tables previously obtained were merged (336 remaining RD entries–59 duplicate entries merged). For the 336 RD entries, a search for correspondence with the Orphanet nomenclature was carried out. A confidence index was introduced to characterize the degree of certainty on the correspondence (High/Medium/Low): 248 matches with a “High” correspondence (74%), 39 matches with a “Medium” correspondence (12%) and 28 matches with a “Low” correspondence (14%) were found. The list was finally reviewed by one of the co-authors, expert on rare diseases (SA), with proposals for modification, grouping or removal of pathologies. An output table including 273 RD entries was finally produced.

Information on each RD (ORPHA number, ICD 10 code, synonyms, inheritance, age of onset and prevalence) was then collected from the Orphanet database for the purpose of producing statistics, and are thus not specific to France (all details can be found in the open-access Orphanet report series). There is a potential bias on the age of onset as the age categories used in the Orphanet database overlap. However, despite potential redundant assignments, this does not call into question the general analysis presented further in the article.

The inheritance codes were simplified in three categories: “Genetic origin” encompasses all diseases with a genetic origin whatever the mode of inheritance. “Partially genetic” includes diseases with a mix of different possible origins, some being genetic, some being acquired. “Non genetic” includes all other diseases, although some of them may have some genetic determinants as minor co-factor. The pathologies were classified by broad categories, following the logics applied in the International Classification of Diseases in its 11th edition (Organization, World Health).

The detailed list of the RDs identified in this work is available in the Supplementary Information section (Supplementary Table S2).

## Results

A total of 273 rare diseases, disorders and conditions were identified as satisfying the criteria of being particularly sensitive to a delayed diagnosis, by loss of opportunity to benefit from appropriate care management options. This list included some infectious diseases (11 RDs) which were not considered further, as posing very different problems. It also included isolated major malformations (9 RDs) which are quite obvious at birth, but also trisomy 21 which is now easily diagnosed, and familial patent arterial duct, which is not posing a diagnostic issue. These conditions were excluded from the analysis as irrelevant in the framework of this project, but all 273 RDs can be found in the Supplementary Material.

**FIGURE 2:**
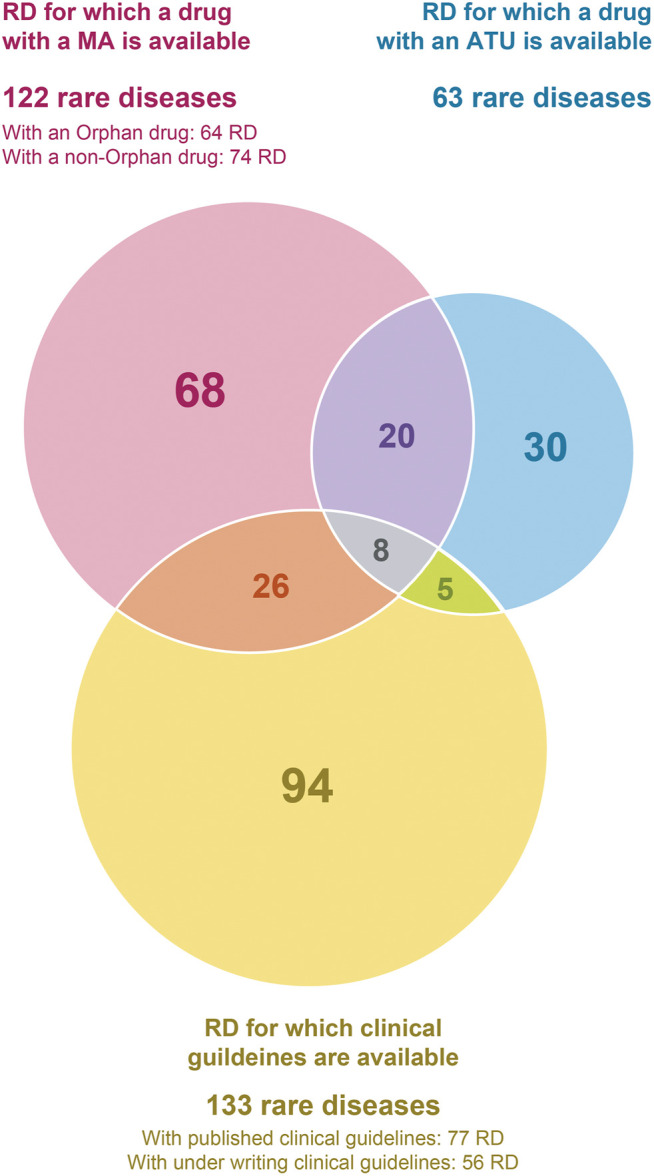
Intersections between the criteria used to select rare diseases for which a delayed diagnosis would be especially detrimental, in the context of the study.

The final list includes 251 conditions, classified in broad categories (Table 1). Notably, most of the conditions identified benefit exclusively either from a drug (118 RDs) or clinical guidelines (94 RDs), while only 39 of them benefit from both (Figure 2). Without surprise, the largest groups are inborn errors of metabolism and multi-systemic diseases, followed by developmental disorders, hematological disorders and neurological disorders. Developmental disorders are well represented because of the large number of clinical guidelines available, despite a small number of drug therapies (Table 2). On the contrary, inborn errors of metabolism rank high because of the large number of marketed drugs, despite a small number of clinical guidelines. In all categories, the number of RDs with both a marketed drug and clinical guidelines is very small (15%).

**TABLE 1:** List of rare diseases for which a delayed diagnosis would be especially detrimental, in the context of the study.

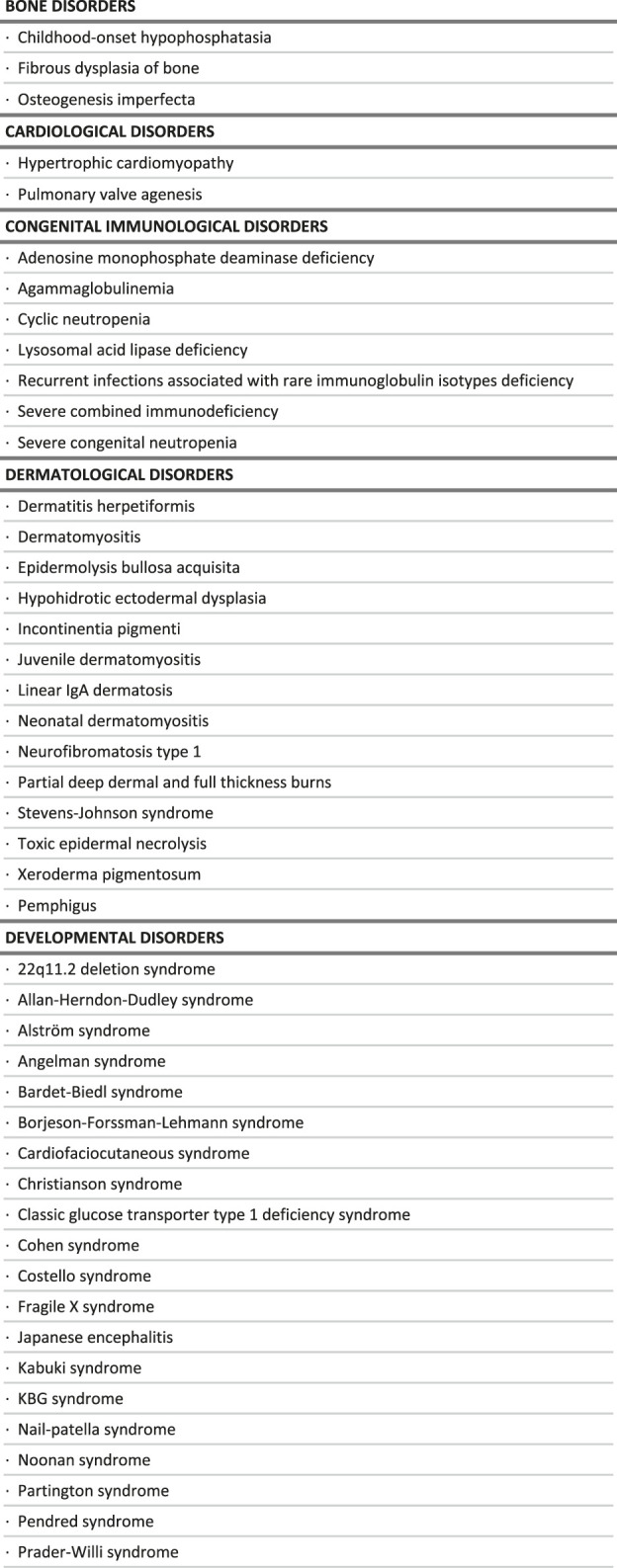
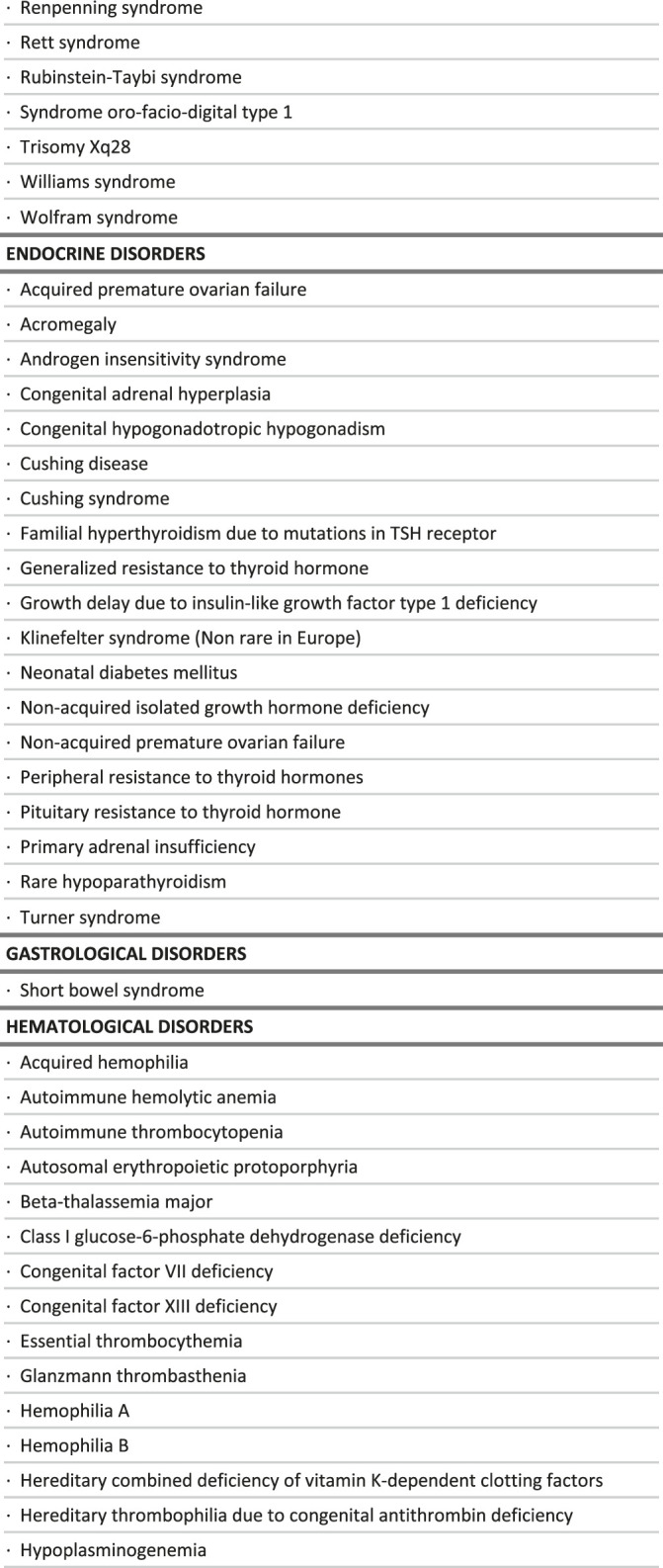
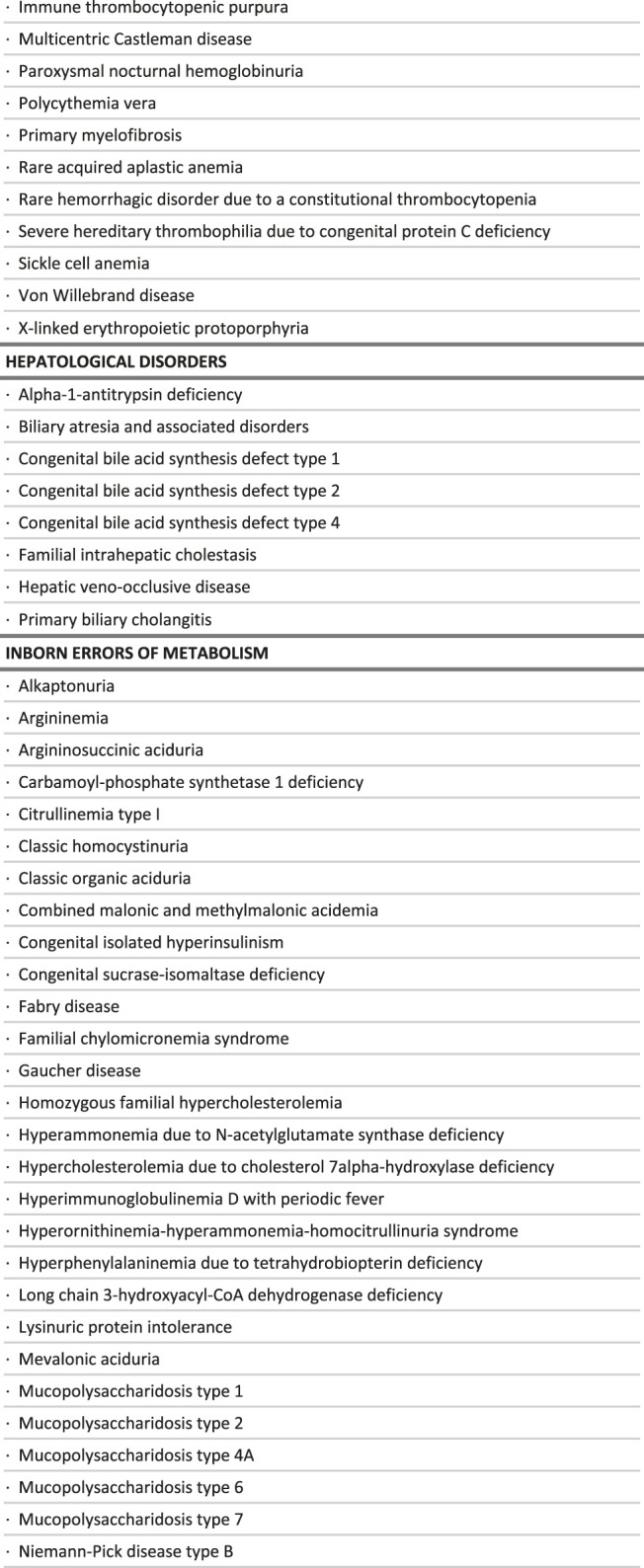
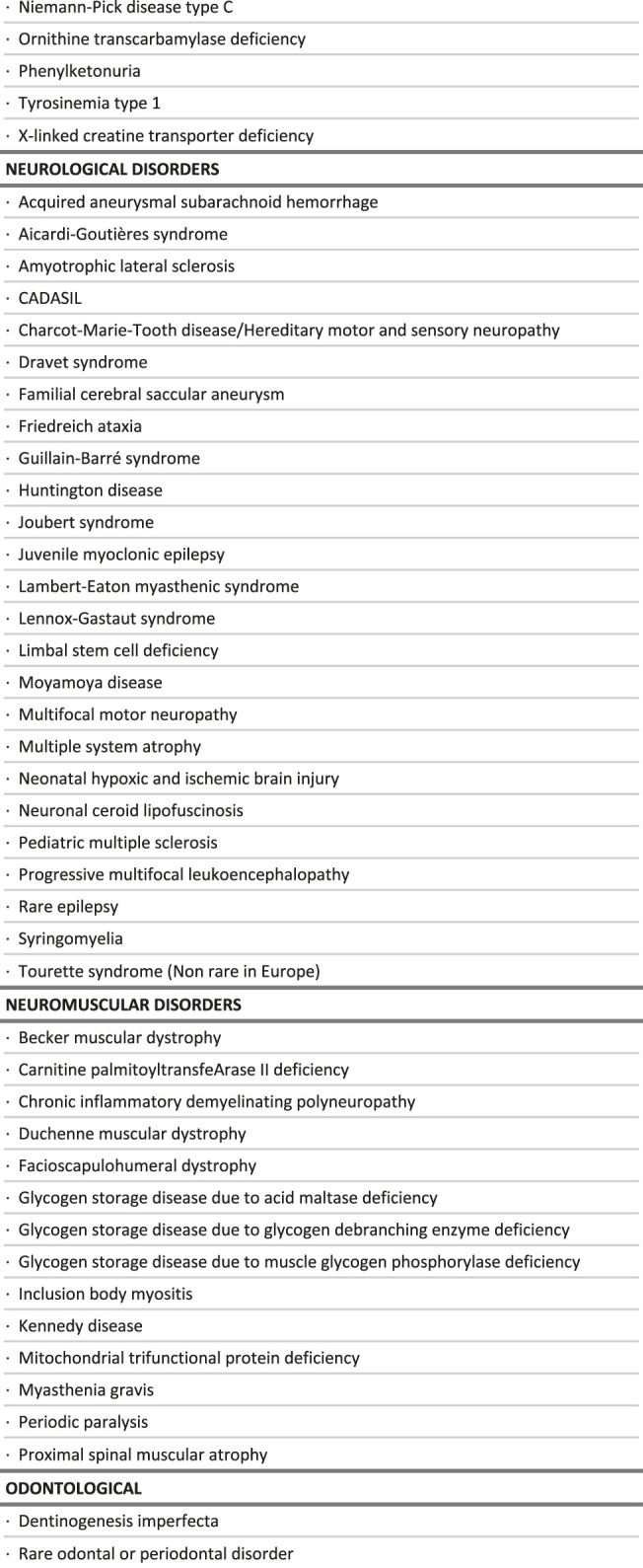
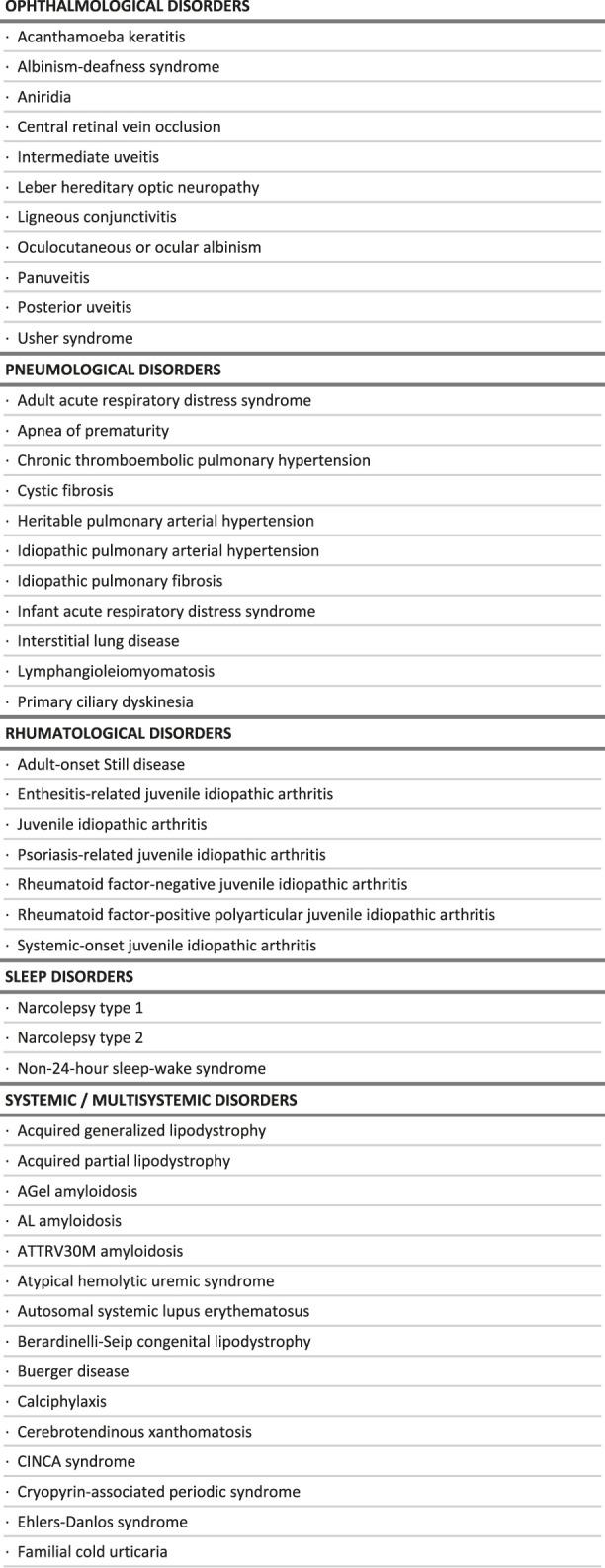
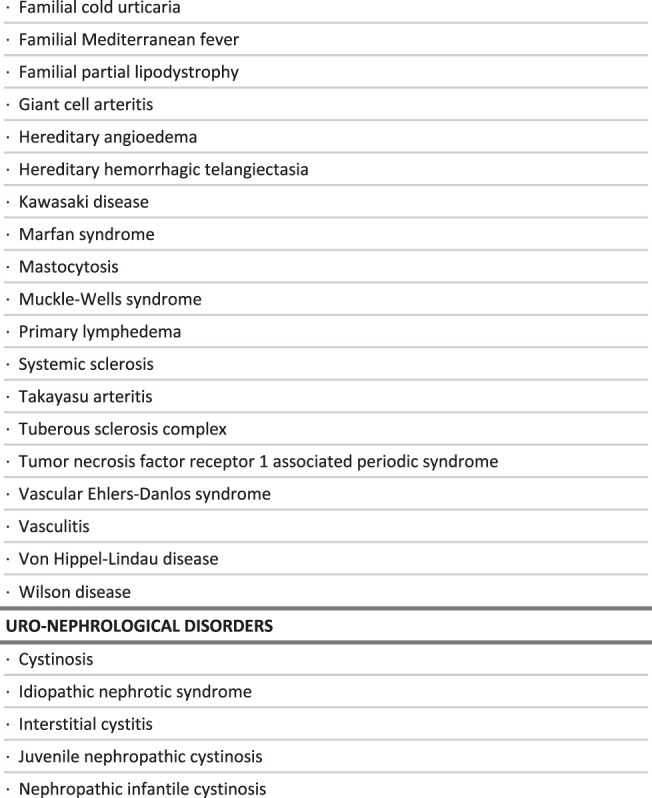

**TABLE 2:** Distribution of care management options by broad groups of diseases.

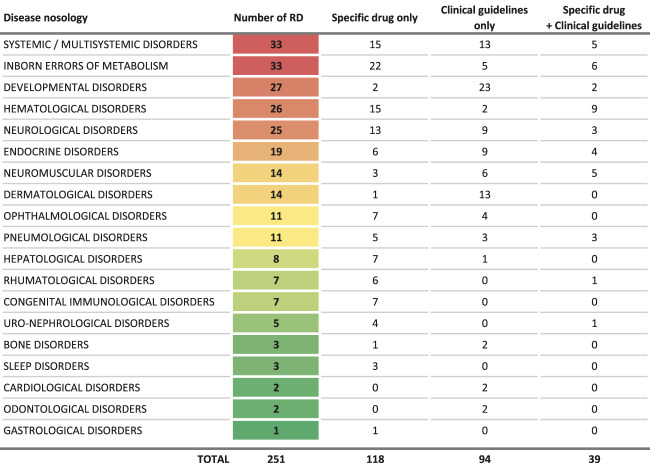

**FIGURE 3:**
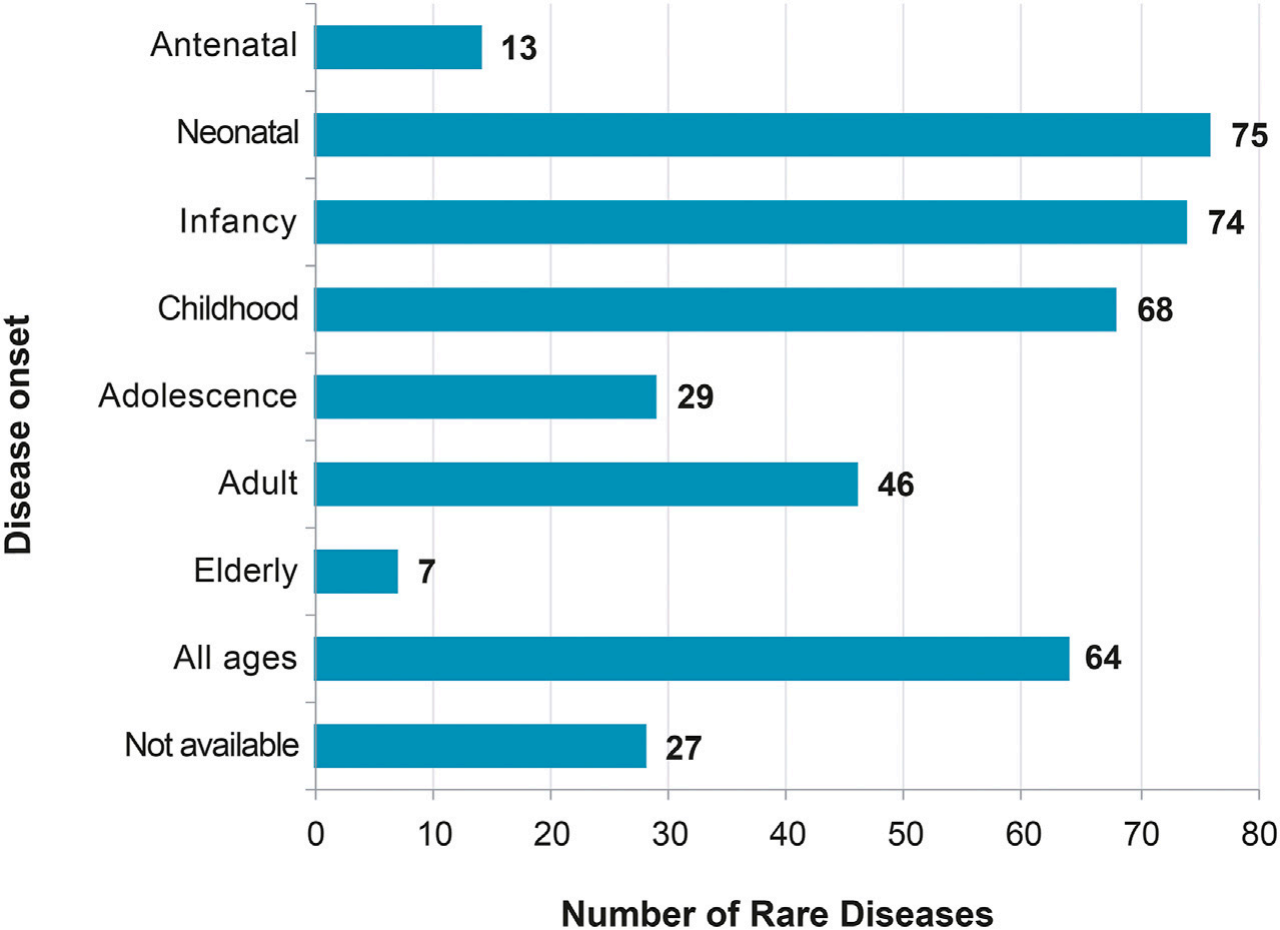
Distribution of the age of onset of the rare diseases for which a delayed diagnosis would be especially detrimental, in the context of the study.

**FIGURE 4:**
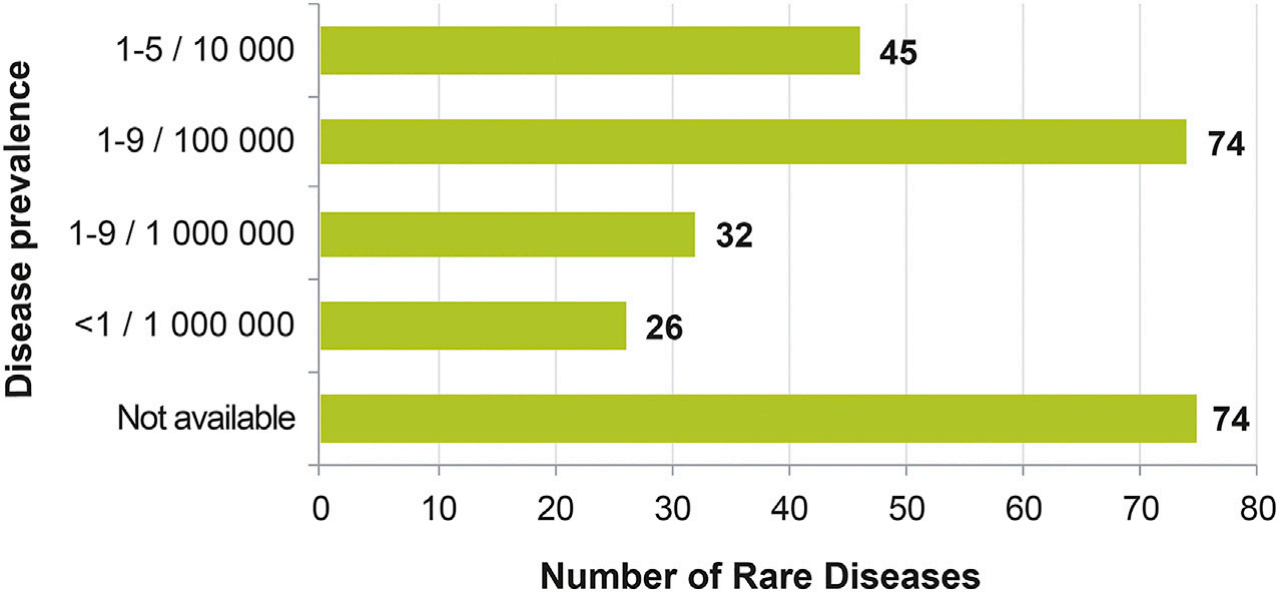
Distribution of the classes of prevalence of the rare diseases for which a delayed diagnosis would be especially detrimental, in the context of the study.

The proportion of RDs in this list with genetic origin is 68.9%, comparable to the 75% for the whole set of RDs in the Orphanet database (Table 3). Moreover, despite a bias in the onset age entry in the Orphanet database (overlap of different entries), most of the 251 conditions are pediatric disorders (Figure 3), which is similar to what is generally described in the RD field. Finally, most of the 251 conditions are very rare (74 RD, 41.8%), or ultra-rare (58 RD, 32.7%), as displayed on the distribution of prevalence (Figure 4).

**TABLE 3:** Distribution of the genetic origin or not of the diseases, by broad groups of diseases.

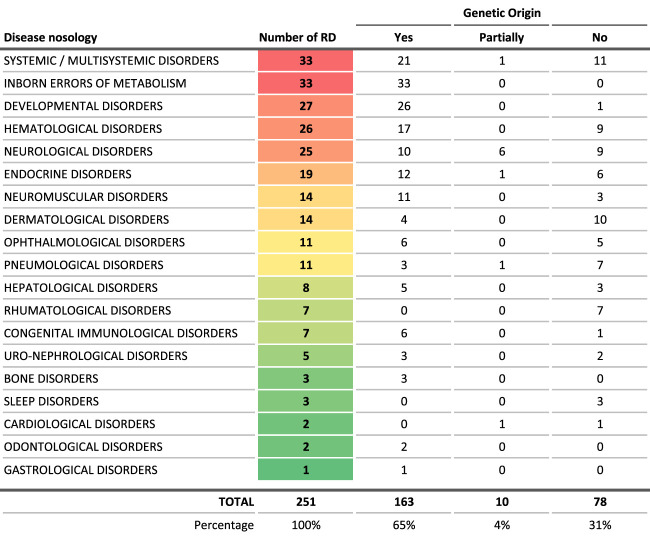

## Discussion

This work explored the feasibility of identifying a subset of RDs at urgent needs for the development of digital-based solutions to reduce the delay to diagnosis. The two proposed criteria for disease selection are based on robust public data. Their use ended in the establishment of an appropriate list of RDs, considering the intended goal. A list ready to be submitted to expert clinicians for validation, before proposing it to potential development and test of both digital and/or organizational solutions. This work could notably be supported by the use of the data from the French National RD Database (BNDMR, 2021) (BNDMR). If the time to diagnosis is deemed not acceptable, this will clearly indicate that digital and/or organizational solutions should be considered in priority for those RDs.

However, even if the study gives relevant results, these sources have *de facto* several limitations. They may suffer from non-completeness. In addition, the dataset is a snapshot of the situation as of January 2018, based on information sources from July 2017 to January 2018. It is representative of the situation at that time only. The proposed list will need to be updated and adapted to the local situation, for any further use.

The work is based on the situation in France, as it was as a proof of concept in the context of a national initiative to develop digital-based solutions for the diagnosis of RDs. Only French clinical guidelines were considered, as the production of these clinical guidelines was a measure of the first and second French National Plan for RDs (PNMR 1, 2004) (PNMR 2, 2010; PNMR 3, 2018). This justifies the choice of this criterion given the scope of this specific study. However, if applied in other countries, other authoritative clinical guidelines could be considered, such as the one from learned societies, national agencies (NHS, 2021) and, in Europe, European Reference Networks. An extension to medical products in clinical trials at European and/or international level could also be considered.

Although not affecting the final list, the proposed grouping of conditions can also be questioned, as the same disease can be considered from several angles, such as the main affected function, the medical specialty caring for patients, the pathophysiology at stake, the etiology, etc. (Rath et al., 2012; Pavan et al., 2017). For this project, it was decided to be as close as possible from the ICD 11 classification system, as it is the most recent attempt to establish an international consensus (Aymé et al., 2015). These choices are however disputable. For example, Neurofibromatosis type 1 is classified as a dermatological disease when it could be also in the developmental anomaly group. Turner and Klinefelter syndrome are considered here as endocrine disorders, when they could also be considered as developmental anomalies. Glucose-6-phosphate dehydrogenase deficiency is in the group of hematological conditions when it could be in the inborn errors of metabolism group. Alpha-1-antitrypsin deficiency is here as hepatological disease and could be a pneumological disease for instance.

Despite these limitations, this study comforts the choice of the two indicators (drugs/clinical guidelines) used for selecting RDs to focus on for the development of digital and/or organizational solutions to improve the time to diagnosis. The two indicators are very differently distributed among the RD groups (Table 3). Most of diseases have, in general, either a specific associated drug or clinical guidelines, while only 39 of them benefit from both (Table 2). The existence of clinical practice guidelines for RDs is, therefore, an independent criterion from the existence of a targeted new therapy, as half of the prioritized RDs in the study has been picked up due to the existence of clinical guidelines only. (Nguengang Wakap et al., 2019).

## Conclusion

The present study aimed at describing an objective methodology to define “priority” RDs for which a delayed diagnosis would be particularly detrimental for the patient.

Identifying such a subset of “priority” RDs would be of great help if and when choices have to be made to develop and test innovative digital or organizational solutions. The proposed approach is robust as it is based on publicly available data. Clarifying choices when taking initiatives to develop solutions, in a field with so many unmet needs, is a requirement for an ethical approach.

Undoubtedly, this preliminary list is to be updated, validated by experts from ERNs for the feasibility to reduce the time to diagnosis, and adapted to local situations, before using it to make decisions.

## Data Availability

Publicly available datasets were analyzed in this study. This data can be found here: https://www.orpha.net/orphacom/cahiers/docs/GB/list_of_orphan_drugs_in_europe.pdf; https://solidarites-sante.gouv.fr/soins-et-maladies/medicaments/professionnels-de-sante/autorisation-de-mise-sur-le-marche/article/autorisations-temporaires-d-utilisation-atu; https://www.ansm.sante.fr/Activites/Autorisations-temporaires-d-utilisation-ATU/ATU-nominative-Liste-des-specialites-autorisees/(offset)/5; https://www.ema.europa.eu/en/medicines/download-medicine-data#rare-disease-(orphan)-designations-section; https://www.has-sante.fr/jcms/c_1340879/fr/protocoles-nationaux-de-diagnostic-et-de-soins-pnds; https://solidarites-sante.gouv.fr/soins-et-maladies/prises-en-charge-specialisees/maladies-rares/article/l-offre-de-soins; https://www.orpha.net/consor/cgi-bin/index.php. Extractions from the databases used, as is at the date of their extraction, can be communicated on request.
